# The Sublingual Gland Flap for Oral Reconstruction: Insights From a Single Institutional Experience

**DOI:** 10.1155/2024/7386967

**Published:** 2024-10-16

**Authors:** Agata Wieczorkiewicz, Joanna Kuczera, Andrei Hramyka, Krzysztof Śliwiński, Jakub Bargiel, Grażyna Wyszyńska-Pawelec, Michał Gontarz

**Affiliations:** Department of Cranio-Maxillofacial Surgery, Jagiellonian University Medical College, Cracow, Poland

**Keywords:** local flap, oral cancer, reconstruction, sublingual gland, sublingual gland flap

## Abstract

**Background:** Following ablative surgery, the reconstruction of oral cavity defects is essential to ensure optimal function and aesthetically acceptable outcomes. The purpose of this study was to retrospectively analyze the effectiveness and complication rates of the sublingual gland flap (SGF) in oral soft tissue reconstruction. The procedure for harvesting SGF and the strengths and limitations of the flap are discussed.

**Materials and Methods:** The study group consisted of 13 patients suffering from oncological diseases who underwent soft tissue reconstruction with SGF. The patient's medical charts were evaluated based on histopathological aspects, postoperative complications, and outcomes. Reconstruction of the floor of the mouth was performed in 8 patients (61.5%) and lower gingiva in 5 patients (38.5%), respectively.

**Results:** Complete epithelialization with closure of the defect was achieved within an average of 2 weeks. The observation period ranged from 1 to 33 months, with an average duration of 11.5 months. Partial flap necrosis and ranula occurred in one patient (7.7%). Furthermore, postoperative bleeding was observed in one patient (7.7%), and wound dehiscence and abscess formation were noted in another (7.7%). Locoregional recurrence of the cancer was observed in one case (7.7%).

**Conclusions:** The SGF is effective for achieving successful reconstruction of small- and medium-sized defects in the lower gingiva and floor of the mouth. The complication rate is relatively low.

## 1. Introduction

Intraoral defects necessitate comprehensive reconstruction to mitigate potential limitations that could adversely affect the patients' quality of life [1]. The procedure is complex and requires the surgeon to possess appropriate skills and experience to achieve postoperative speech and swallowing function, restore dental occlusion, prepare for dental rehabilitation, and attain the best overall aesthetic result after the reconstruction. The reconstruction can be quite difficult, time-consuming, and often results in suboptimal outcomes, particularly when the patient is treated for advanced oncological disease [2]. The primary cause of intraoral soft tissue defects is commonly cancer, particularly squamous cell carcinoma (SCC). Surgical flaps are typically classified according to their pattern of vascularity, which can be either random or axial, and their proximity to the primary defect, which can be local, regional, or free. Each of the flaps has specific indications for use, although they can often be combined with each other to cover a large defect [3]. Currently, free flaps are frequently used in head and neck reconstruction [3, 4]. However, local flaps have many advantages, and they can be a great alternative to free flaps in the case of reconstructing small- to medium-sized soft tissue defects [5]. The decision regarding which flap to use is made on an individual basis, as certain flap types are more advantageous than others in specific cases, for certain patients, tumors, and reconstruction sites [3–5].

The sublingual gland flap (SGF) is a reconstructive option for intraoral defects that is not widely used by surgeons. In addition, information on this flap is limited in the literature. The SGF can be a stand-alone reconstructive option for oral defects as well as an option to line deeper aspects of defects reconstructed with other flaps. The sublingual glands are the smallest of the three major salivary glands of the oral cavity, weighing 2–4 g, which are located under the mucous membrane of the anterior floor of the mouth, superior to the mylohyoid muscle, deep to the sublingual folds, opposite the lingual frenulum, and lateral to the genioglossus muscle and mandible [1]. The gland flap's accessible location facilitates harvesting and subsequent use to cover soft tissue defects. Blood supply is provided by the submental and sublingual arteries and branches of the lingual and facial arteries, accompanied by veins which drain blood from the glands [1]. Rich vascularization decreases the risk of flap necrosis. The lingual nerve and submandibular Wharton's duct are located close to the gland, which makes its preparation meticulous.

Due to the small size of the sublingual gland, the flap is indicated to repair only small- to medium-sized soft tissue defects (<4 cm) [6]. Larger defects are more complex and usually require the use of free flaps or a combination of different flaps. The use of the SGF is a reliable technique for the reconstruction of anterior and lateral defects of the floor of the mouth, as well as of the adjacent gingiva, following the ablation of T1 or T2 tumors [6].

The aim of the study was a retrospective analysis of the effectiveness of SGF in the oral cavity soft tissue reconstruction. Furthermore, a detailed description of the surgical technique was provided, along with a literature review.

## 2. Materials and Methods

### 2.1. Patients

**Figure 1:**
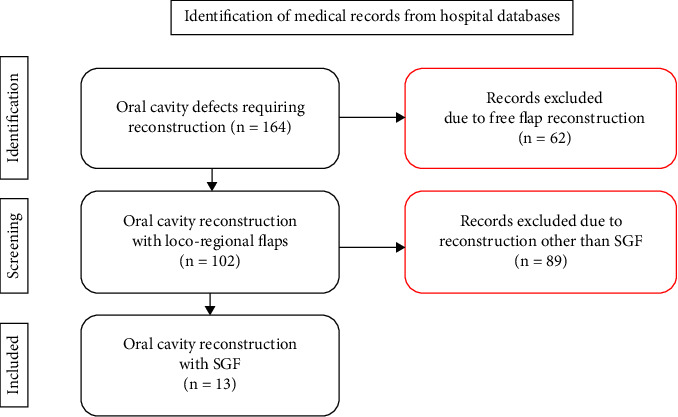
Flowchart of the process of establishing the study group. Identification of medical records from hospital databases.

A retrospective analysis of 164 medical records of oral cavity defects requiring flap reconstruction at the Department of Cranio-Maxillofacial Surgery, University Hospital in Cracow, Poland, was conducted over the period from December 2020 to March 2024. Free flap reconstructions and non-SGF locoregional flap reconstructions were excluded from the study. Only patients with SGF reconstruction were considered eligible for analysis. The process of identifying the study group is presented in a graphical format in Figure 1.

The study group consisted of 13 patients (8 males and 5 females) with oral cancer and precancerous lesions who underwent intraoral soft tissue defect reconstruction with SGF. The average age of patients was 61.7 years. The patient's medical charts were evaluated based on clinical manifestations, histopathological aspects, postoperative complications, recurrences, follow-up, and overall outcomes. This study was approved by the Institutional Review Board of the Jagiellonian University (no. 1072.6120.229.2021). Due to the retrospective nature of the research and because only medical records were obtained, the review board approved the study without requiring patient consent, provided that all personal information was kept confidential. All patients suffered from oral cancer or precancerous lesions (erythroplakia and leucoplakia). In each case, a frozen section examination of the sublingual gland was carried out immediately before the SGF placement in order to verify the absence of cancerous infiltration.

### 2.2. Surgical Technique

**Figure 2:**
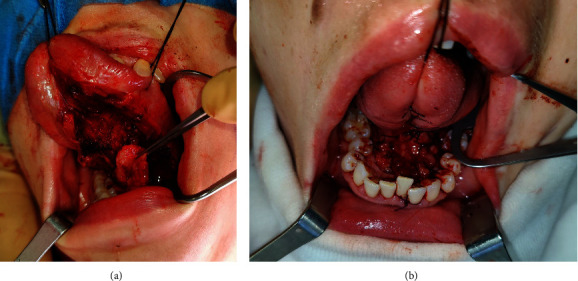
Surgical technique in a 37-year-old patient suffering from SCC of the floor of the mouth. (a) Defect after radical excision of the cancer from the floor of the mouth with exposure and harvest left SGF. (b) Suturing of the flap and mucosa.

All patients underwent surgical procedures under general anesthesia in a supine position. The initial phase of the procedure involved the radical excision of the pathological lesion with an adequate clear margin. The subsequent procedure involved the exposure of the sublingual gland. The gland was carefully separated from the mylohyoid muscle and sublingual mucosa, ensuring the preservation of both the lingual nerve and Wharton's duct. The major sublingual duct was ligated and divided. Furthermore, in order to ensure the optimal blood supply to the SGF, it was essential to preserve the sublingual and submental arteries and veins. Following the intraoperative frozen section examination of the sublingual gland, the flap could be harvested and rotated into the surgical defect. The final step was suturing the SGF and mucous membrane with 4.0 Vicryl or Monocryl sutures (Figure 2).

In one of the cases, intubation of the Wharton's duct was carried out to keep patency for saliva flow and avoid sialadenitis of the submandibular gland. All patients with a histopathologic diagnosis of SCC (76.9%) also underwent elective, selective bilateral neck dissection at Levels I–III. In the postoperative period, patients were fed through a nasogastric tube for the next 10–14 days. They were instructed to maintain good oral hygiene and not to smoke cigarettes to avoid infection and ensure proper wound healing. Sutures were removed 10–14 days after surgery. Patients were followed up in an outpatient clinic once a month.

## 3. Results

**Figure 3:**
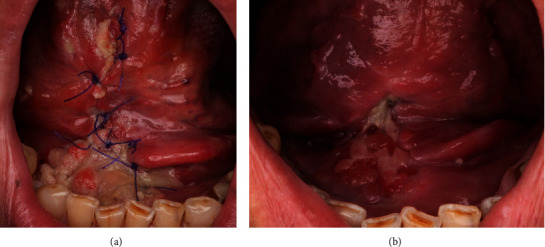
Wound healing process by granulation in a 56-year-old patient. (a) 3 days after surgery. (b) 10 days after surgery.

Reconstruction of the floor of the mouth was performed in 8 patients (61.5%) and lower gingiva in 5 patients (38.5%). In 5 patients (38.5%), a flap from the left sublingual gland was used, in 3 patients (23%) from the right gland, and in 5 patients (38.5%) from both. The postsurgical wound has been healed by secondary intention (edges did not approximate). The whole wound was filled by granulation tissue matrix, clinically visible usually as a white or gray coating (Figure 3). This healing process was desirable for its ability to repair lost tissue and restore function and aesthetics. Complete epithelialization with closure of the defect was achieved in 2 weeks on average.

**Figure 4:**
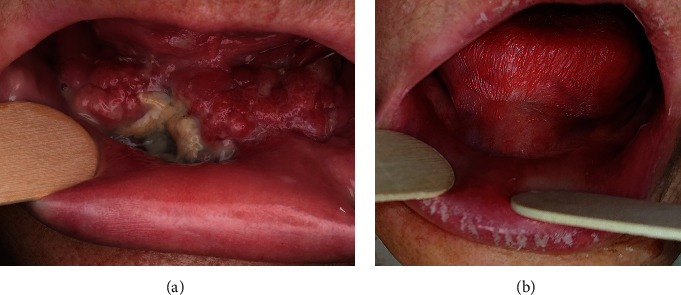
A 74-year-old female patient after bilateral SGF reconstruction. (a) Partial necrosis of the right flap 3 days after surgery. Clinically, granulation of SGF may mimic SCC. (b) Clinical follow-up of 6 months after surgery and adjuvant radiochemotherapy with significant xerostomia.

Postoperative complications were observed in 4 patients (30.8%) (Figure 4). In 2 cases, adjuvant treatment such as radiotherapy or radiochemotherapy was required due to nodal metastasis. Only one patient (7.69%) had a locoregional recurrence of SCC 1 year after surgery. The mean follow-up time was 11.5 months (ranging from 1 to 33 months). The clinical characteristics of the study cohort with outcomes are shown in Table 1.

## 4. Discussion

The SGF for oral defect reconstruction presented in this study provided good aesthetic and functional results. In cases of soft tissue reconstruction of the floor of the mouth or lower gingiva, minor disorders of tongue mobility were observed, such as deviation to one side and difficulty in protrusion forward. However, all patients were satisfied with the results. Speech, chewing, and breathing were maintained. The use of SGF to reconstruct soft tissue defects in the oral cavity is not popular among maxillofacial surgeons. This reluctance is probably due to the many advantages and disadvantages of this flap [6].

Placement directly under the mucous membrane of the floor of the oral cavity, combined with easy and quick exposure of the gland, as well as very good blood supply through branches of the lingual and facial arteries, which prevents flap necrosis, are certainly the most important advantages of this reconstructive option. This is a local flap adjacent to the defect, so there is no need for additional incisions to harvest it, which means no additional scars after healing, especially extraoral scars. Even if part of the sublingual gland has been resected to achieve clear margins after oral cancer excision, the remaining part of the gland can be harvested and used for reconstruction. It can be easily placed into the defect during a one-stage procedure performed by a team of surgeons. Harvesting time for SGF is approximately 10 min, and once healed, its color and structure closely resemble the surrounding tissue, making it nearly indistinguishable. Furthermore, sublingual neoplasms are rare, accounting for less than 1% of salivary gland tumors, making this gland safe to use [7]. After the operation, the patient does not require a long hospital stay and the healing process is fast.

One of the main limitations of the SGF is that it is not suitable for large defects, which are usually reconstructed with free and pedicled musculomucosal flaps or fasciocutaneous flaps, or a combination of these [4–6, 8–10]. The range of rotation and mobility of SGF is also limited. Only the lower part of the oral cavity can be reconstructed with SGF. This disadvantage is not observed in the island FAMM flap, which can be rotated and tunnellized under the mandible to reconstruct the lower and upper parts of the oral cavity [9]. However, the harvesting time of the island FAMM flap is longer than that of the SGF. In addition, the close proximity of the lingual nerve and submandibular Wharton's duct requires caution during gland dissection to avoid damaging these structures. Other disadvantages of SGF are associated with the potential risk of certain postoperative complications. Ranula is the most common pathology of the sublingual glands comprising an extravasation of saliva from the gland due to trauma of the Rivinus duct [11, 12]. Fortunately, since the SGF is rotated and sutured into the oral cavity, only oral ranulas are observed, which can be easily treated by excision or marsupialization [11, 12]. Another complication may be ankyloglossia, a condition characterized by limited tongue mobility due to abnormalities associated with scarring after surgical treatment. However, postoperative ankyloglossia can be successfully treated by secondary surgery with local plasty or mucous membrane transplantation [13, 14]. Another problem is that granulation and prolonged secondary healing for up to 1 month can mimic the local recurrence of SCC (Figure 3(a)). This problem is not observed with other local intraoral flaps such as FAMM, Bozola, or tongue flaps used for oral cavity reconstruction since they contain mucosa and in most cases heal with primary intention [5, 8–10, 15, 16]. Secondary healing with epithelialization of the flap is also typical of the buccal fat pad flap. The secondary healing process in the buccal fat pad flap is unpredictable and may cause excessive scarring with local tissue contracture and persistent trismus [17]. Another technical problem is the difficulty in suturing the SGF properly to the remaining mucosa close to the teeth in the floor of the mouth. In these patients, there is a higher risk of wound dehiscence and local inflammation. In this study, excessive intraoral bleeding was also observed in one case 3 days after surgery, which was the need for surgical intervention. This complication may be a result of the good blood supply to the sublingual gland, which is not covered by the mucosa, and any masticatory trauma or extensive tongue movement may cause bleeding from the flap.

Due to the small number of cases in the cohort presented, a review of the literature was performed to verify the suitability of the use of SGF and the rate of complications. The English language literature available in PubMed, Web of Science, Embase, and Google Scholar databases from January 2000 to March 2024 was reviewed to identify articles related to the SGF. The search was performed using the key words: SGF, local flaps, and intraoral reconstruction. Only 4 papers on SGF use were included in the review (Table 2) [1, 6, 18, 19]. According to the literature review, between 2000 and March 2024, SGF was used for reconstruction in 96 patients with a mean age of 63 years. The most common indication for SGF reconstruction was oral cancer (91.7%), followed by mandibular exposure due to osteomyelitis (6.2%). The most common site of reconstruction was the lower gingiva (33.3%), followed by the floor of the mouth (31.2%) and the ventral part of the tongue (28.1%). Complications were observed in 9 patients (9.4%), mainly oral ranula and ankyloglossia, indicating good flap adaptation and adequate reconstruction of lost soft tissue. The rate of complications in SGF is comparable with FAMM flap where partial and total necrosis of the flap and other complications can be found in 12.2%, 2.9%, and 12.8%, respectively [20].

### 4.1. Study Limitations

This analysis aims to present the utility of SGF in small- and medium-sized defects of oral cavity reconstruction; however, it has its limitations. The main limitation was the small size of the study group. This small group is the result of the infrequent use of the SGF due to the limitations of this flap. Defects of the oral cavity more often require free flaps or larger local or regional flaps for reconstruction. In addition, statistical analysis was not performed due to the small sample size. However, a comparison of harvesting time, hospital stay, and complication rate among SGF, FAMM, and soft tissue free flap reconstruction can be the perspective study.

In conclusion, the SGF is effective for achieving successful reconstruction of small and medium-sized defects in the lower gingiva and floor of the mouth. The main indication for SGF reconstruction is defects after oral cancer excision followed by mandibular exposure due to osteomyelitis. The complication rate is relatively low and most commonly manifests as oral ranula and ankyloglossia.

## Figures and Tables

**Table 1:** Clinical characteristics of patients with sublingual gland flap reconstructions of the oral cavity.

Case	Age/sex	Site	Clinical stage	Histopathology	Adjuvant treatment	Local recurrence	Complications	Follow-up (months)
1	69/M	FOM	T1N0M0	SCC G1	No	No	No	27
2	81/M	Lower gingiva	T1N0M0	SCC G2	No	Yes	No	33
3	49/M	FOM	40 mm	Erythroplakia	No	No	No	20
4	49/F	Lower gingiva	T4N0M0	SCC + CGCL	No	No	No	14
5	74/F	Lower gingiva	T2N3bM0	SCC G3	PORT + CTH	No	Oral ranula and partial flap necrosis	14
6	53/M	FOM	15 mm	Leukoplakia	No	No	No	8
7	78/F	Lower gingiva	T2N0M0	SCC G2	PORT	No	Ankyloglossia with speech impairment	11
8	37/F	FOM	T1N0M0	SCC G2	No	No	No	8
9	56/M	FOM	T1N0M0	SCC G1	No	No	Bleeding	4
10	67/M	FOM	T2N0M0	SCC G1	No	No	No	4
11	63/M	Lower gingiva	T4N0M0	SCC G1	No	No	No	3
12	75/M	FOM	T1N0M0	SCC G2	No	No	No	2
13	51/F	FOM	T3N0M0	AdCC	No	No	Wound dehiscence, abscess	1
Total	Mean: 61.7 years M: 8 (61.5%) F: 5 (38.5%)	FOM: 8 (61.5%) Lower gingiva: 5 (38.5%)	Stage I: 5 Stage II: 2 Stage III: 1 Stage IV: 3	SCC: 10 (77%) AdCC: 1 (8%) PL: 2 (15%)	Yes: 2 (15%) No: 11 (85%)	Yes: 1 (8%) No: 12 (92%)	Yes: 4 (31%) No: 9 (69%)	Mean: 11.5 months

Abbreviations: AdCC, adenoid cystic carcinoma; CGCL, central giant cell lesion; CTH, chemotherapy; F, female; FOM, floor of the mouth; M, male; PL, premalignant lesion; PORT, postoperative radiotherapy; SCC, squamous cell carcinoma.

**Table 2:** Reconstruction of intraoral defects using the sublingual gland flap: a literature review.

First author/year of publication	Years	No. of cases	Age (average)	Histopathology no. (%)	Localization no. (%)	Complications no. (%)
Malinge/2021 [6]	2006–2016	72	No data	SCC 72 (100%)	FOM: 22 (30%) Lower gingiva: 18 (25%) Tongue: 25 (35%) Other: 7 (10%)	Bone exposure: 2 (2.8%) Ranula: 1 (1.4%) Ankyloglossia: 1 (1.4%)

Eguchi/2018 [18]	2017-2018	4	70–87 (77.25)	SCC: 3 (75%) VC: 1 (25%)	Tongue: 2 (50%) Lower gingiva: 2 (50%)	Ranula 1 (25%)

Jose/ 2021 [1]	2018-2019	6	45–71 (62.0)	MRONJ: 4 (66.6%) OML: 1 (16.7%) ORN: 1 (16.7%)	Lower gingiva: 6 (100%)	None

Benech/2021 [19]	2021	1	78	SCC 1 (100%)	Lower gingiva: 1 (100%)	None

Present study	2020–2024	13	37–81 (61.69)	SCC 9 (69.2%) Erythroplakia: 1 (7.7%) Leucoplakia: 1 (7.7%) CGCL + SCC: 1 (7.7%) AdCC: 1 (7.7%)	FOM: 8 (61.5%) Lower gingiva: 5 (38.5%)	Ranula: 1 (7.7%) Flap necrosis: 1 (7.7%) Bleeding: 1 (7.7%) Abscess: 1 (7.7%) Ankyloglossia: 1 (7.7%)

Total	2006–2023	96	37–87 (63.0)	Oral cancer: 88 (91.7%) Osteomyelitis: 6 (6.2%) PL: 2 (2.1%)	Lower gingiva: 32 (33.3%) FOM: 30 (31.2%) Tongue: 27 (28.1%) Other: 7 (7.4%)	Ranula: 4 (4.2%) Bone exposure: 2 (2.1%) Ankyloglossia: 2 (2.1%) Flap necrosis: 1 (1%) Bleeding: 1 (1%) Abscess: 1 (1%)

Abbreviations: AdCC, adenoid cystic carcinoma; CGCL, central giant cell lesion; FOM, floor of the mouth; MRONJ, medication-related osteonecrosis of jaws; OML, osteomyelitis; ORN, osteoradionecrosis; PL, precancerous lesions; SCC, squamous cell carcinoma; VC, verrucous carcinoma.

## Data Availability

The data used to support the findings of this study are available from the corresponding author upon reasonable request.
